# Plant Responses to Herbivory, Wounding, and Infection

**DOI:** 10.3390/ijms23137031

**Published:** 2022-06-24

**Authors:** Salma Mostafa, Yun Wang, Wen Zeng, Biao Jin

**Affiliations:** College of Horticulture and Plant Protection, Yangzhou University, Yangzhou 225012, China; dh18033@yzu.edu.cn (S.M.); mz120190969@yzu.edu.cn (Y.W.); mx120200764@yzu.edu.cn (W.Z.)

**Keywords:** airborne signaling, epigenetic regulation, intracellular signaling, physical barrier, plant–herbivore interaction, secondary metabolite, signal transduction

## Abstract

Plants have various self-defense mechanisms against biotic attacks, involving both physical and chemical barriers. Physical barriers include spines, trichomes, and cuticle layers, whereas chemical barriers include secondary metabolites (SMs) and volatile organic compounds (VOCs). Complex interactions between plants and herbivores occur. Plant responses to insect herbivory begin with the perception of physical stimuli, chemical compounds (orally secreted by insects and herbivore-induced VOCs) during feeding. Plant cell membranes then generate ion fluxes that create differences in plasma membrane potential (*V*m), which provokes the initiation of signal transduction, the activation of various hormones (e.g., jasmonic acid, salicylic acid, and ethylene), and the release of VOCs and SMs. This review of recent studies of plant–herbivore–infection interactions focuses on early and late plant responses, including physical barriers, signal transduction, SM production as well as epigenetic regulation, and phytohormone responses.

## 1. Introduction

Plant–insect interactions can be divided into two main types: mutualistic interactions (with pollinating insects) and antagonistic interactions (with herbivores) [1]. Many studies have examined antagonistic interactions between plants and herbivores to understand plant defensive mechanisms. Plants use versatile defensive strategies during sudden biotic attacks, involving both physical and chemical barriers. The primary physical barriers include epidermal layers, hairs, thorns, and trichomes. When a plant comes into contact with an insect, chemical changes occur. The plant’s initial chemical response to an insect attack is cell wall modification [2], in which signals released by the insect are received by receptors that activate the plant’s immune system. Following herbivore attacks, plants are often susceptible to infection by pathogens (e.g., bacteria, fungi, and viruses). At the infection site, cell surface receptors recognize molecular attacks and activate pattern-triggered immunity (PTI) [3]. The host cell and extracellular spaces are exposed to secretions of different effector proteins from herbivores, which activate effector-triggered immunity (ETI) [3]. Thereafter, mobile signals are generated and migrate to distal tissues, activating the secondary immune system. This complex regulatory mechanism is known as systemic acquired resistance (SAR) [4].

Several changes occur in the plant cell plasma membrane following herbivore attack. The response begins with the perception of molecular stimuli and effector proteins, prompting increased cytosolic calcium ([Ca^2+^] _cyt_) levels, the depolarization of plasma membrane potential (*V*_m_) [5], and the activation of mitogen-activated protein kinases (MAPK), which are responsible for protein phosphorylation [6], nicotinamide adenine dinucleotide phosphate (NADPH) oxidase activation, and reactive oxygen species (ROS) and reactive nitrogen species (RNS) production. Afterwards, phytohormones including jasmonic acid (JA), salicylic acid (SA), and ethylene are rapidly activated and produced in large amounts. These phytohormones are basic components of stress-related signaling pathways [7,8]. Plants also accumulate secondary metabolites (SMs), including volatile organic compounds (VOCs), as a late defense response [5]. SMs act as feeding deterrents and toxins and reduce the nutritional value of plant food [1].

Biotic stresses also induce epigenetic changes at the DNA and histone levels. Genetic and epigenetic regulation are important for organism growth and maintenance, as well as survival under unfavorable conditions [9]. Through DNA methylation, histone modification, and small non-coding RNAs, these regulatory processes have significant impacts on plant resistance and signal adjustment. Several recent studies have described these mechanisms in response to biotic stresses, particularly DNA methylation [10].

Humanity is facing challenges related to sustainable food security, and biotic stress is considered a major cause of crop losses [11]. Accordingly, a better understanding of plant defensive mechanisms under biotic stresses could help avoid future crop losses. In this review, we summarize recent progress and discoveries related to plant–herbivore–infection interactions, focusing on physical and chemical barriers, plant defense signaling, and the role of epigenetic regulation in the early response to insect attack, as well as the production of SMs and VOCs during the late response.

## 2. Plant Defense Prior to Herbivore Attack

### Plant Physical Barriers

**Figure 1:**
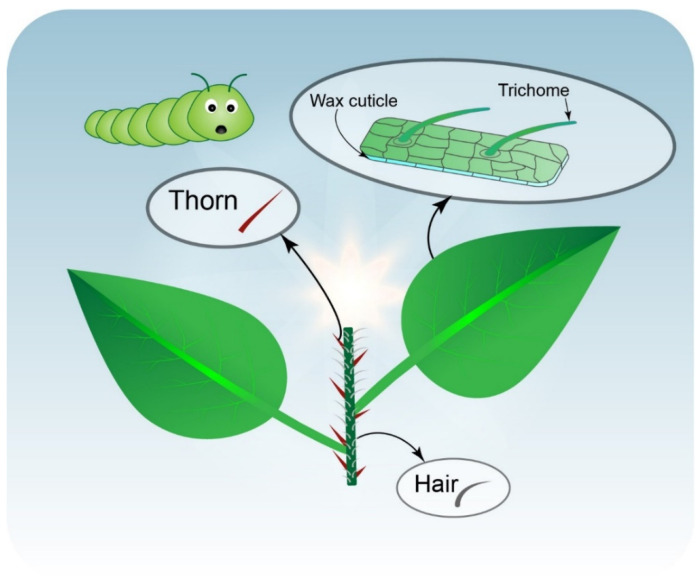
Schematic diagram of plant physical barriers against herbivory attack, including wax cuticle layers, trichomes, thorns, and hairs.

Plant morphological and microstructural properties provide protection against herbivore attacks. These natural physical barriers, which act as the first line of defense against insect herbivory, include hairs, trichomes, spines, thorns, and cuticles covering aerial plant parts (Figure 1) [12,13]. Plant cuticular waxes play a major role in the regulation of plant–insect interactions at multiple levels [13]. For example, in ant-plants (*Macaranga griffithiana*; Euphorbiaceae), cuticular waxes are slippery due to their high triterpenoid content, which protects ants against other insects [14]. Transgenic *Gossypium hirsutum* plants, with a higher wax content, are more resistant against whiteflies (*Bemisia tabaci*) [15]. Plants with higher numbers of spines are more resistant to insects; for example, *Manduca sexta* caterpillars grew faster in three Solanum species (*Solanum carolinense*, *Solanum atropurpureum*, and *Solanum aethiopicum*) with their spines experimentally removed than in plants with intact spines [16].

Trichome structures, including glandular trichomes, increase plant resistance by influencing insect oviposition and/or feeding [17]. High trichome density further increases plant resistance against attacks. For example, strawberry plant resistance to the two-spotted spider mite (*Tetranychus urticae*) was increased at higher trichome densities [18]. Both the physical structure of trichomes and their chemical constituents influence plant protection [19]. Trichomes mainly consist of compounds with low nutritional value for insects, such as cellulose and phenolics, which can be toxic to insect herbivores [19]. Interestingly, non-glandular trichomes in maize and soybean plants contain silicon, which improves plant resistance against fall armyworm (AW; *Spodoptera frugiperda*) [20]. Glandular trichomes also act as reservoirs for terpenes, fatty acid derivatives, and VOCs, some of which can attract natural enemies that protect host plants indirectly [21]. Previous studies have focused on elucidating the roles of plant physical structures in defense [12,13]. In addition, genetic engineering to develop transgenic plants with enhanced physical barriers may also improve plant resistance against herbivore attacks.

## 3. Plant Responses during Herbivore Attack, Wounding, and Infection

### 3.1. Signal Transduction in Host Plants

Interactions between plant signal transduction and insect feeding play a significant role in the induction of plant immunity [22]. During herbivore attacks, plant cell surface-localized pattern recognition receptors (PRRs) enhance plant immunity through the recognition of plant-derived damage-associated molecular patterns (DAMPs), microbe-associated molecular patterns (MAMPs) [23], herbivore-associated molecular patterns (HAMPs) [24], and phytocytokines and the activation of PTI against pathogens [23]. Generally, PRRs consist of a short trans-membrane domain attached to a varying extracellular domain and conserved cytoplasmic kinase [25]. HAMPs are induced by chemical cues found in herbivore oral secretions (OSs) or oviposition fluid [24]. However, very few PRRs for HAMPs have been described [25]. Herbivore attacks induce changes in plasma membrane potential (*V*m), followed by the generation of secondary messengers (e.g., [Ca^2+^] _cyt_) and ROS [24], as well as a rapid increase in phytohormones (e.g., JAs) [26]. These changes are among the earliest plant defense responses, occurring within seconds to minutes after an herbivory attack [5]. HAMP compounds also provoke the release of leaf volatiles and terpenoids [25]. For example, ROS are induced in tomato (*Solanum lycopersicum*) protoplasts in response to tobacco hornworm caterpillar (*Manduca sexta*) OS [24], and OSs from two pest species, *Helicoverpa armigera* and *Spodoptera litura*, prompt rapid induction of JA signaling in cotton plants (*Gossypium hirsutum*) [26].

Leaf damage caused by insect attacks suggests the delivery of resistance elicitors through attachments to different receptors on the plasma membrane [5]. The interaction between resistance elicitors and receptors causes a fluctuation in *V*m, i.e., cell membrane electrical potential maintained by ion flux balance across the plasma membrane [27]. These electrical signals move from cell to cell in plants, carrying different messages. Unbalanced ion fluxes give rise to positive or negative changes in *V*m, defined as depolarization and hyperpolarization, respectively [27], which induce plant defense responses. For example, the herbivore *Spodoptera littoralis* induces *V*m depolarization in infected Lima bean plants (*Phaseolus lunatus*) [28].

The involvement of Ca^2+^ in plant responses upon herbivore attack has been demonstrated in several studies. Herbivore wounds cause massive increases in Ca^2+^ cytosolic ion content following *V*m changes [5]. Ca^2+^ influx is regulated by protein channels and transporters located in the plasma membrane and Ca^2+^ sensors [5]. Many Ca^2+^-binding proteins and calcium sensors in plants recognize Ca^2+^ signals and regulate downstream responses. Calcium sensors carry one or more EF-hand Ca^2+^-binding motifs [29]. The main sensor families are calmodulins (CaMs)/calmodulin-like proteins (CMLs), Ca^2+^/CaM-dependent protein kinases (CCaMKs), calcium-dependent protein kinases (CDPKs/CPKs), calcineurin B-like protein (CBL), and CBL interacting protein kinase (CIPK) modules [29,30].

CaMs are highly conserved calcium-modulated proteins that consist of two globular domains. Each domain contains two EF-hand motifs, and one motif can bind one Ca^2+^ ion. Therefore, one CaM molecule can bind four Ca^2+^ ions [31]. Several CaM-binding transcription factors (TFs) have been shown to be involved in defense responses through crosstalk with plant hormones [32]. In infected Arabidopsis plants, the CaM-binding TF AtSR1/CAMTA3 functions as a negative regulator of SA through the interaction of SA-related genes [33]. CaM is also essential to the function of the CaM-binding protein CBP60g in Arabidopsis infected by *Pseudomonas syringae*; CBP60g increases SA levels following pathogen/biotic attacks. The disconnection between CBP60g and CaM prevents SA production, demonstrating the role of CaM in plant defense [34].

Other divergent forms of CaM include CMLs, 50 members of which have been discovered in Arabidopsis [35]. In soybean, the overexpression of CMLs (SCaM-4/-5) improves resistance to a wide range of insects and pathogens (e.g., bacteria, fungi, and viruses) [32,36]. Other CMLs are specific to plant immunity and biotic stress, such as CML42 and CML43 [5,37]. The downregulation of Arabidopsis CML42 enhances resistance to *Spodoptera littoralis*, which is correlated with the upregulation of JA-related genes [38]. By contrast, CML37 acts positively in plant defense against *S. littoralis*, suggesting opposing functions in plant resistance [32,39]. Still other CMLs (e.g., CML9, 11, 12, 16, 17, and 23) are involved in insect attack resistance, and are highly regulated in plants treated with lepidopteran herbivore OS [32,40].

Interestingly, although CCaMKs have been detected in various plants, including legumes, maize (*Zea mays*), and tobacco, they are not present in Arabidopsis [41]. Unlike CaMs, CCaMKs possess a CaM-binding domain and visinin-like domain (Ca^2+^-binding domain) with three EF-hands. CCaMKs also have an autoinhibitory domain overlapping the CaM-binding domain [41,42]. However, the role of CCaMKs during herbivore attacks has been poorly studied, in contrast to their role in abiotic stress responses. Further study is required to obtain a thorough understanding of CCaMK functions in the regulation of Ca^2+^ ions, particularly in plant biotic attacks.

CPK sensors act as multipurpose proteins, whereas a single CPK protein functions in Ca^2+^ binding and signaling for phosphorylation [5]. Recently, the genes *AcoCPK1*, *AcoCPK3*, and *AcoCPK6* were found to enhance plant resistance against *Sclerotinia sclerotiorum* in pineapple, *Ananas comosus* [43]. CPKs are also involved in the regulation of K^+^ channel transportation in Arabidopsis [44].

After the detection of Ca^2+^ signals by CBL proteins, CBL and CIBK sensors interact to form CBL–CIBK complexes; the CBL–CIBK signaling pathway is regulated by complex mechanisms in association with other signaling pathways [5]. ROS signaling molecules regulate Ca^2+^ signals upon biotic stress. For example, *AtCIPK6* overexpression negatively regulates ROS production in Arabidopsis. Thus, downregulation of *AtCIPK6* increased plant resistance to the pathogen *Pseudomonas syringae* [45]. Interactions among MeCIPK23, MeCBL1, and MeCBL9 and their overexpression increase the cassava (*Manihot esculenta*) defense response to *Xanthomonas axonopodis* pv. *manihotis* [46]. When AtCIPK26 interacts with AtCBL1 or AtCBL9, the NADPH oxidase AtRBOHF is phosphorylated, and ROS accumulate through the action of RBOHF [47]. CBL–CIBK complexes also play a role in K^+^ regulation; K^+^ deficiency is correlated with the accumulation of JA [48] and ROS and the activation of Ca^2+^ to enhance plant defensive mechanisms [47]. Under low-K^+^ conditions, AtCBL1 and AtCBL9 activate the phosphorylation of Arabidopsis K^+^ transporter 1 (AKT1) through the action of AtCIPK23 [47]. CBL10 also competes with CIPK23 for binding with K^+^ channels (AKT1). The binding of CBL10 with AKT1 hinders AKT1-mediated K^+^ flux into the cytoplasm [49].

**Figure 2:**
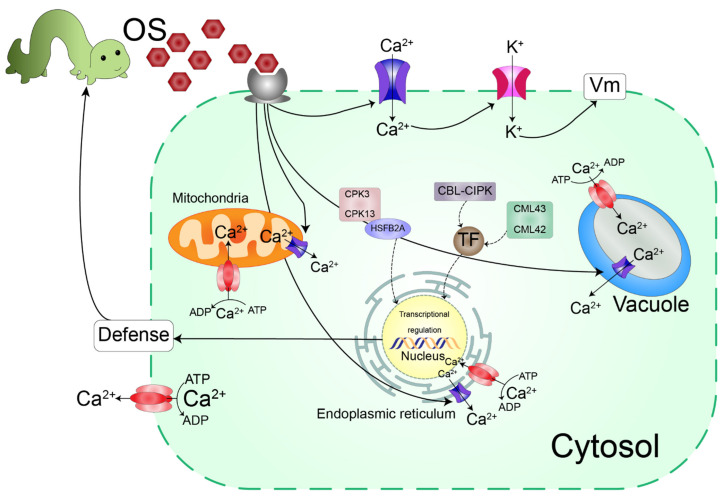
Schematic diagram of the calcium signaling pathway following biotic attack. After an attack, resistance elicitors are released in herbivore oral secretions (OS), precipitated, and bound to receptors, causing a rapid increase in calcium cytosolic ([Ca^2+^] _cyt_) content. Calcium channels and ATP-dependent Ca^2+^ pumps in the cell membrane and cell organelles (e.g., mitochondria, vacuoles, and endoplasmic reticulum) organize Ca^2+^ ions inside and outside the cell/organelles. Greater increases in Ca^2+^ ions trigger potassium (K^+^) channel activation, causing plasma membrane potential (*V*m) depolarization. Different calcium receptors (e.g., CBL–CIPK, calcineurin B-like protein- CBL interacting protein kinase, CML42/CML43, calmodulin-like proteins 42/43, and CPK3/CPK13, calcium-dependent protein kinases3/13) increase to activate transcription factors, such as HSFB2A. Finally, transcriptional regulation in the nucleus induces plant herbivore defense (Modified from [5]).

ATPases, which are the main energy source for plant cells, also play an essential extracellular role in plants under stress. Extracellular ATP signaling is associated with secondary messengers, such as Ca^2+^, ROS, and NO [50]. Ca^2+^-ATPases act as ion transporters across cellular membranes. Ca^2+^-ATPases belong to the P-type ATPases superfamily, which is generally divided into two groups according to ATPase localization in plant cells: P-IIA ER-type Ca^2+^-ATPases (ECAs) and P-IIB autoinhibited Ca^2+^-ATPases (ACAs). ECAs are analogous to animal sarcoplasmic–endoplasmic reticulum Ca^2+^-ATPases, whereas ACAs are equivalent to animal PM-type ATPases [30,51]. ACAs are more selective, transporting only Ca^2+^, whereas ECAs also transport Cd^2+^, Mn^2+^, and Zn^2+^ [51]. Calcium signaling pathways following herbivore attack are illustrated in Figure 2.

Salivary proteins induce plant–insect interactions through ROS accumulation and cell death enhancement. The elevation of intracellular Ca^2+^ ions and ROS act as signal transducers under biotic stress. Several forms of ROS are present in plants, including hydrogen peroxide (H_2_O_2_•), superoxide anion (O_2_−•−), hydroxyl radical (HO•), peroxynitrite (ONOO), and singlet oxygen (1O_2_) [27]. ROS are mainly generated through the action of NADPH oxidase, which is activated by Ca^2+^ ions, producing O_2−_ for further conversion to H_2_O_2_ in the plasma membrane [52]. For example, salivary protein 1 (NlSP1) in the brown plant hopper (BPH) induces an immune response in rice plants through H_2_O_2_ aggregation and cell death [53]. Salivary proteins also produce crosstalk between ROS and plant phytohormone signals. For example, high SA concentrations induce ROS production, whereas SA affects the metabolism of ROS in mitochondria. SA plays an important role in plant defense against biotic attacks and is essential for SAR formation [52]. ROS production induces SA accumulation and vice versa; however, SA also promotes ROS scavenging [54,55].

The exact mechanism of RNS in plants remains ambiguous; however, nitric oxide (NO) is involved in plant stress tolerance and acts as a signaling molecule during herbivore attacks [56]. In *Pisum sativum* (pea), NO accumulates in response to feeding by the aphid *Acyrthosiphon pisum* [57]. Exogenous NO application to infected pea plants induces a defense mechanism against aphids, thus reducing their growth [58]. Dynamic interactions between ROS and the NO signaling pathway have been suggested during abiotic stresses [59], although whether such crosstalk occurs during biotic stress remains unclear. Further study is needed to elucidate the role of RNS during biotic stress in plants.

**Figure 3:**
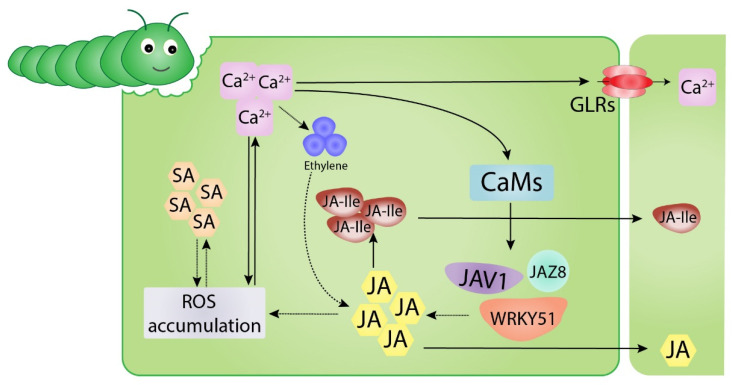
Schematic diagram of the interactions among reactive oxygen species (ROS), Ca^2+^, and phytohormones (JA, jasmonic acid, SA, salicylic acid, and ethylene) within the cell upon biotic attack. Ca^2+^ influxes activate ethylene and calmodulins (CaMs) of JAV1, dismantling the JAV1–JAZ8–WRKY51 (JJW) complex to activate JA biosynthesis and thereby producing jasmonoyl-L-isoleucine (JA-Ile). High JA concentrations cause ROS accumulation, which in turn induces SA and vice versa. Distal transfer of Ca^2+^ (through glutamate receptor-like (GLR) channels), JA, and JA-Ile occurs between cells.

JA is an important plant regulator involved in plant responses to wounding. Changes in JA and phytohormone levels are induced by the conversion of Ca^2+^ ions, ROS, and RNS signaling. However, the relationship between ROS production and phytohormone signaling is complex. For example, high JA levels induce ROS accumulation, whereas low JA levels induce NO, which is antagonistic to ROS activation [60]. In tomato plants, JA loss leads to ROS accumulation in response to fatty acid amide elicitation [55]. The involvement of Ca^2+^-binding proteins in JA regulation has been demonstrated in wounded plants, where calcium influx triggers the activation of Ca^2+^/CaM-dependent phosphorylation of JAV1, dismantling the JAV1–JAZ8–WRKY51 (JJW) complex to activate JA biosynthesis [61] (Figure 3). However, the role of Ca^2+^-binding proteins in phytohormone regulation remains poorly understood.

### 3.2. Intracellular Signaling

The release of long-distance signals from the site of damage to different plant parts is an important defensive strategy necessary for plant survival. Systemic signaling is involved in the communication between wounded and unwounded plant tissues [27]. Once cell membrane and signal transduction are interrupted, ion channels move ions across plasma membranes, facilitating long-distance communication between cells. The transfer of these signals from wounded sites to other cells induces a defense response [27]. For example, the non-selective glutamate receptor-like channels (GLRs) recently discovered in Arabidopsis are involved in Ca^2+^ signaling transmission during herbivory. Both Arabidopsis and *Solanum lycopersicum* GLRs are involved in long-distance signaling [62]. In addition to Ca^2+^ signaling, electrical signals, ROS, and crosstalk between ROS and Ca^2+^ have large impacts on long-distance signaling (reviewed in [62]).

Previous studies have demonstrated that JA, methyl jasmonate (MeJA), and jasmonoyl-L-isoleucine (JA-Ile; the bioactive form of JA) can be transferred through both phloem and xylem from wounded tissues, accumulating up to several centimeters from distal unharmed tissues [63,64,65]. JA is also synthesized in vascular bundles [66,67,68] such that damage to veins results in high JA and JA-Ile accumulation [64]. The wounded site of the leaf causes JA activation in both harmed and unharmed tissues, indicating the translocation of JA from wounded to unwounded sites [69]. As demonstrated in Arabidopsis, shoot wounding induces the relocation of endogenous JA through phloem tissues and the translocation of cis-12-oxo-phytodienoic acid (OPDA), the precursor of JA and its derivatives, leading to the conversion of JA into JA-Ile to initiate JA signaling in unharmed roots [69]. Deuterium-labeled analogs have been used in *Nicotiana tabacum* (tobacco) and *Solanum lycopersicum* (tomato) plants. Exogenous application of both JA and JA–Ile induced high accumulation thereof in distal leaves, in both control plants and wounded plants untreated with exogenous JA or JA–Ile, whereas the mobility of JA–Ile was greater than that of JA [70] (Figure 3).

However, some studies have shown that JA/JA-Ile induction in distal intact tissues following plant wounding was derived from de novo biosynthesis, rather than transport from the damaged site [71]. In tomato plants, enzymes such as lipoxygenase (LOX) and allene oxide synthase (AOS; involved in JA synthesis) are localized in the companion cell–sieve element complex of vascular bundles, in addition to accumulated JA and OPDA, demonstrating JA biosynthesis in these tissues [66,72]. However, other studies have rejected the hypothesis that JA is resynthesized in distal tissues following herbivore attacks, suggesting that phytohormone distribution is dependent on vascular connections between leaves, as JA concentrations increase in both locally damaged and systemically unharmed leaves. The JA precursor OPDA is not systemically induced; its concentration increases only in local tissues after continuous wounding [73,74]. These findings suggest that increases in JA concentrations do not occur through de novo biosynthesis [65]. Further study is required to improve our understanding of the transport of JA and its derivatives between cells, as well as long-distance signaling over long distances far from damaged sites.

### 3.3. Airborne Signaling

VOCs are emitted by plants in response to mechanical damage or herbivore feeding [75,76]. Long-distance signaling by plants through the release of VOCs elicits systemic immunity against herbivore attacks [65,75]. Generally, VOCs are induced by cell disruption; thereafter, they function as DAMPs and HAMPs, through which plants recognize damage and herbivore attacks [77]. These VOCs are synthesized de novo after damage; molecules such as oligosaccharines and peptides are then generated as secondary signals [78]. For example, the peptide precursor IbHypSys is highly induced following wounding of sweet potato plants, leading to the formation of sporamin, an important defense protein [79]. *Spodoptera exigua* caterpillar OS strongly induces VOCs in cotton plants in response to DAMPs and HAMPs [80]. VOC emission patterns can be divided into two main types; the first occurs within a few seconds after damage (e.g., enzymes in leaf tissues), and the second within hours after damage (e.g., several types of terpenes and phenolic compounds) [78]. Some stored terpenes are emitted directly upon tissue damage, including pre-existing secretory structures [78]. Other phenomena that arise in response to damage include the accumulation of SMs, such as phenolic compounds and tannins [81,82,83], the activation of defensive oxidative enzymes by MeJA or ethylene [84], and the high expression and biosynthesis of proteinase inhibitor genes [85,86]. In addition, a high concentration of VOCs leads to the repellence of biotic attack. For instance, *Sitophilus granarius* L. and *Tribolium confusum* are repelled by the highest concentrations of cereal VOCs [87,88].

## 4. Plant Response to Biotic Attack through Epigenetic Regulation and SMs

### 4.1. Epigenetic Regulation

Epigenetic regulation plays a crucial role in plant resistance and signal adjustment in response to herbivore attacks. DNA methylation is involved in plant immunity and regulates chromatin structure, DNA stability, and gene expression [89]. In *Brassica rapa*, methylation changes are induced in both leaves and flowers following leaf damage by *Pieris brassicae* caterpillars [90]. Soybean plant resistance responses to soybean cyst nematodes result in DNA methylation [91]. Recently, CHH methylation was reported to regulate defense responses against the fungal pathogen *Blumeria graminis* f. sp. *tritici* in infected wheat plants [92].

The function of small RNAs (sRNAs) has been demonstrated in plant–herbivore interactions. In plants, sRNAs are divided into two major classes according to their roles, i.e., microRNAs (miRNAs), which are produced from single-strand stem-loop precursor structures, and short interfering RNAs (siRNAs), which are derived from double-strand RNA transcripts [93,94]. Both miRNAs and siRNAs, individually or in combination, can improve plant resistance against diseases [95]. In the tea plant *Camellia sinensis* L., 130 known and 512 novel miRNAs were identified in response to attacks by the geometrid *Ectropis oblique* [96]. In rice plants, 464 known and 183 novel miRNAs were identified after brown planthopper (BPH) attacks [97]. In sweet potato, the target genes of miR408 (*IbKCS*, *IbPCL*, and *IbGAUT*) are highly expressed in plants after wounding, whereas their expression is suppressed in transgenic lines overexpressing miR408, confirming the participation of miRNAs in plant defense [98].

Long non-coding RNAs (LncRNAs) are involved in several developmental and biological processes, such as chromatin reshaping and transcriptional activation [99], contributing to plant defenses against biotic attacks [100]. For example, lncRNAs have been detected in *Nicotiana attenuata* in response to herbivore attacks, and the accumulation of lncRNAs induced the release of active JAs [100]. Interaction has been observed between herbivores and lncRNAs, and AW-elicited lncRNAs have been identified in treated plants [101]. LncRNAs have also been studied in rice plants infected with blast fungus, confirming their role in defense [102]. In infected cotton plants, differentially expressed lncRNAs are involved in resistance to *Verticillium dahliae* disease [103], and lncRNAs are involved in plant resistance to aphid damage [104].

Histone modifications, such as acetylation, methylation, and ubiquitination, which occur at histone N-terminal tails, have been demonstrated in plant–pathogen interactions [105]. Histone acetylation levels are affected by the activity of histone acetyltransferases (HATs) and histone deacetylases (HDACs). HATs connect the acetyl moiety of acetyl-CoA to lysine amino groups and are usually correlated with increased gene expression, whereas HDACs detach acetyl groups from histones, causing gene suppression [106,107]. Both complex subunits of HATs, ELONGATOR PROTEIN2 (ELP2) and ELP3, enhance plant defenses through their acetyltransferase activity [108]. In Arabidopsis, the involvement of HATs (AtELP2 and AtELP3) and HDACs (AtHDA6, AtHDA9, AtHDA19, AtSRT2, and AtHD2B) in plant resistance against pathogens has been demonstrated in several studies [107,109,110,111,112]. Plant cell wall acetylation status affects plant resistance to phloem-feeding insects [2]. Pectin acetylesterase 9 (PAE9) enhances DAMP-induced defenses against phloem-feeding aphids in Arabidopsis [2]. In wheat, the HAT complex TaGCN5-TaADA2 plays a role in the regulation of cuticular wax biosynthesis in response to powdery mildew [113]. In rice, C-terminal tail binding of subunit OsRpp30 to the HDAC OsHDT701 causes a negative defense response to the fungal and bacterial pathogens *Magnaporthe oryzae* and *Xanthomonas oryzae*, respectively [114]. Tomato plant resistance to bacterial wilt (*Ralstonia solanacearum*) in two different cultivars showed that differential HDAC expression led to the downregulation of resistant genes [115].

Histone (de)methylation positively (negatively) regulates immunity defense-associated genes in plants [108]. For example, H3K4 and H3K36 methylation activates the transcription of defense-related genes, whereas H3K9 and H3K27 methylation causes gene repression [105,116]. Previous studies have identified histone methyltransferases in Arabidopsis (AtATX1, AtSDG8, and AtSDG25), in addition to histone demethylases (AtJMJ27 and AtIBM1), which are involved in regulating plant–pathogen interactions [105,117,118]. The Arabidopsis SET DOMAIN GROUP methyltransferase (SDG8) plays a role in immunity. The SDG8 mutant exhibits increased susceptibility to *Alternaria brassicicola* and *Botrytis cinerea*, activating JA-responsive genes and promoting misregulation of MKK3 and MKK5, which are protein kinases involved in the phosphorylation cascade activated upon pathogen attack [119,120]. The rice demethylase JMJ705 contributes to the regulation of defense responses, whereas its overexpression reduces H3K27me2/3 levels, leading to the upregulation of defense-related genes and the enhancement of plant resistance to pathogens [121].

The regulatory mechanism of histone ubiquitination controls the interaction of target proteins with other proteins [122]. Histone ubiquitination occurs as a result of adding one or more ubiquitin groups to lysine residues of target proteins through the action of various enzymes [123]. There are two major types of ubiquitination, mono- and polyubiquitination, distinguished according to the number of ubiquitin chains attached to the target proteins. Polyubiquitination of the 26S proteasome, an ATP-dependent, multi-subunit protease complex, causes the degradation of target proteins, whereas monoubiquitination acts as an endogenous signal and does not cause the degradation of target proteins [124]. For example, in Arabidopsis, monoubiquitination components, such as histone monoubiquitination1 (HUB1), are involved in plant defenses against necrotrophic fungal pathogens [125]; for example, H2B monoubiquitination (H2Bub) is involved in the defense against *Verticillium dahliae* toxins [122]. The Arabidopsis polyubiquitination component UBC22 is involved in plant defense against pathogens [126]. JA accumulation after a herbivore attack leads to the binding of COI1, a component of the ubiquitin E3 ligase SCFCOI1 and first receptor of JA-Ile, to JAZ proteins; their reaction with 26S proteasomes causes the ubiquitination and degradation of JAZ repressors, leading to the release of TFs of JA-regulated genes [8,127]; this demonstrates the relationship between JA and histone ubiquitination.

### 4.2. SMs

In plant–insect interactions, chemical changes are induced in the host plant, such as increased production of SMs, which are involved in the regulation of plant resistance against herbivores. SM concentrations vary among compounds and alter the metabolite profile of the infected plant [128]. Five classes of SMs play a role in the regulation of plant defense: glucosinolates, benzoxazinoids, aromatics, terpenes, and green-leaf volatiles [129]. For example, conifers release accumulated terpenes (monoterpenes) in response to bark beetle attacks [130]. Herbivore behaviors in response to released VOCs are highly variable. During ecological interactions, herbivores can be attracted to volatiles emitted at low or moderate concentrations, which serve as ecological cues. By contrast, herbivores are repelled by other volatiles produced by heavily infested plants [131].

Phenolic compounds, including lignin, coumarins, furanocoumarins, flavonoids, and tannins, are also highly produced following plant infection and play a role in plant defense strategies [132]. Meta-analyses of plants infected by beneficial microbes, pathogens, or insects have confirmed increased phenolic compound levels [133]. Lignin contributes to both biotic and abiotic stress tolerance. During herbivore attacks, lignin acts as a physical barrier to herbivory, toughening plant tissues and thus rendering them non-digestible to insects and other herbivores [134]. Coumarins are secreted by plant roots and are mainly involved in iron uptake; they also play a role in plant protection against herbivores and infections [135]. For example, coumarins isolated from *Artemisia granatensis* inhibited herbivory by insects such as *Spodoptera littoralis*, *Myzus persicae*, and *Rhopalosiphum padi* [136]. Furanocoumarins are toxic compounds released by few plant species as a defense against herbivory, mainly in Apiaceae and Rutaceae species [134]. For example, some hogweed plant species (Apiaceae) produce high concentrations of furanocoumarins, protecting plants against insect feeding [137]. Flavonoid compounds are important SMs produced by plants [138] and accumulate in high amounts after herbivore attacks. In tea leaves (*Camellia sinensis*), herbivore attacks cause upregulation of flavonoid-related genes, leading to flavonoid accumulation and inducing a defense response against the tea green leafhopper [139]. Tannins, which are anti-nutritional compounds, are also induced following attack, to reduce the nutritional quality of plant tissues for insects [140]. In plant–insect interactions, tannins build complexes that reduce the amount of nitrogen, thereby preventing insects from hydrolyzing proteins by inhibiting their digestive enzymes [141]. However, some insects possess tannin-binding salivary proteins that may reduce the negative effects of tannins [140].

Lectins are proteins found naturally in most plants; they enhance plant immunity during plant–insect interactions through the release of cytokines and other effectors. However, the mechanism of plant lectin production in response to insect or pathogen attacks remains unclear [142].

Sulfur-containing compounds, including glutathione, glucosinolates, phytoalexins, and defensin, play an important defensive role in plants. Glutathione participates in several detoxification reactions in plants, in addition to its signaling regulation role in plant–herbivore interactions [143]. For example, in soybean plants (*Glycine max* L. Merr.) infected with the nematode *Heterodera glycines*, glutathione mediated the generation of H_2_O_2_. Low concentrations of glutathione increase H_2_O_2_ levels, thereby reducing nematode accumulation [144]. Many glucosinolates have been identified in nearly all parts of the plant. The active forms of glucosinolate compounds accumulate in response to damage by herbivores. Glucosinolate concentrations influence *Brassica rapa* plant resistance against *Delia radicum* insects [145]. Phytoalexins are low-molecular-weight compounds with antimicrobial effects; they accumulate as part of the defense against insect attacks [146]. For example, diterpenoid phytoalexins are highly concentrated in maize (*Zea mays*) attacked by the European corn borer (*Ostrinia nubilalis*) [147]. Defensins have antimicrobial, antifungal, and insecticidal properties in plants and are induced by pathogen attacks [148]. The role of defensins in defense is to inhibit insect digestive enzymes, such as α-amylase and proteases [148].

Nitrogen-containing compounds work effectively in plant defensive mechanisms. Alkaloids, cyanogenic glycosides, and non-protein acids are the main nitrogen-containing compounds. Alkaloids are accumulated in plants under different stresses. Pyrrolizidine alkaloids (PAs) are toxic compounds that defend plants against insect herbivory; the most effective forms of PAs are jacobine and erucifoline [149]. For example, jacobine causes high thrip mortality, demonstrating its significant role in plant protection [150].

**Figure 4:**
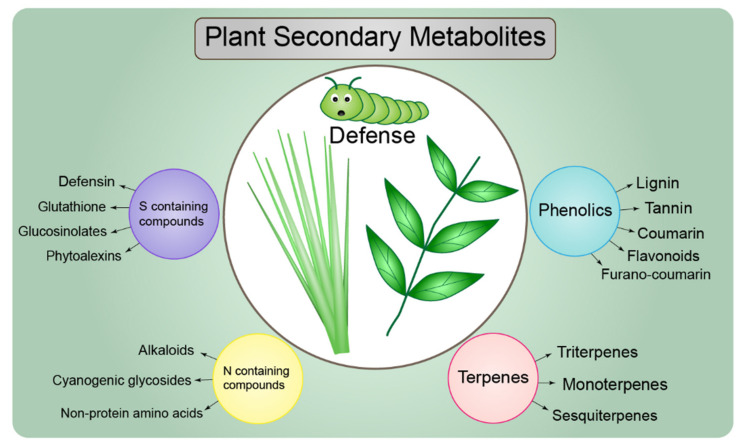
The main plant secondary metabolites (SMs) involved in plant defenses against herbivores, broadly classified as phenolics, terpenes, and sulfur (S)- and nitrogen (N)-containing compounds.

Cyanogenic glycosides are important chemical components in plant defense mechanisms. In cassava (*Manihot esculenta*), feeding by the whitefly (*Bemisia tabaci*) activates cyanogenic glycosides, whereas the resulting hydrogen cyanide is converted to beta-cyanoalanine [151]. A role of non-protein acids has been demonstrated in many studies. Several forms of non-protein acid compounds have been studied in the context of plant defense, such as γ-aminobutyric acid (GABA), β-aminobutyric acid (BABA), and canavanine [152]. BABA inhibits aphid growth on tic bean plants (*Vicia faba* L. var minor) [153]. GABA tends to increase under biotic stresses, as reported in Arabidopsis leaves upon herbivory. GABA accumulation is stimulated after wounding by insect feeding (*Spodoptera littoralis*) [154,155]. The production of toxic canavanine is mainly limited to Fabaceae species. Seeds of *Canavalia*, *Dioclea*, *Hedysarum*, and *Medicago sativa* L. are rich in canavanine [156]. Some insect herbivores (e.g., *Drosophila* species) avoid feeding on plants containing canavanine, demonstrating its toxicity and repellent effects against insects [157]. Plant SMs involved in defense are listed in Figure 4.

JA and its derivatives increase the production of SMs after herbivore attacks by inducing related biosynthetic enzymes [158]. The artificial application of these phytohormones (e.g., JA, MeJA, and MeSA) induces SM accumulation. For example, in *Bidens pilosa*, the application of MeJA and MeSA as resistance elicitors enhances SM biosynthesis (e.g., caffeoylquinic acids, tartaric acid esters, chalcones, and flavonoids) [159]. Economically, SMs are one of the main factors influencing crop quality and yield. An improved understanding of the relationship between SM induction and herbivore attacks may lead to more comprehensive pest management and thus higher yield production [131].

## 5. Conclusions and Future Perspectives

Plant resistance to biotic attacks is one of the important survival abilities to overcome ecological challenges. Globally, biotic attacks are considered one of the major reasons for crop loss; therefore; humanity is confronted by obtaining a sustainable food security. In this review, we summarized plant responses to herbivory and infection, focusing on physical barriers, signal transduction, epigenetic regulation, and SMs. Upon biotic attack, plants employ physical barriers (e.g., hairs, thorns, and wax layers) and SMs (including VOCs) as a defensive arsenal. During herbivory attacks, plants initiate signal transduction to activate a variety of defensive mechanisms that are mainly mediated by phytohormones (e.g., SA and JA), Ca^2+^, and ROS, as well as gene expression and epigenetic regulation. Recent advances in molecular biology and metabolomics have accelerated fundamental research on plant–herbivore interactions. Large numbers of metabolites, enzymes, and genes are involved in plant defense responses. These studies characterized plant defense pathways, thus improving our understanding of the mechanisms underlying plant defense responses. However, several unresolved issues remain, which warrant further investigation.

The molecular basis of plant response pathways under pathogen infection has received extensive research attention, whereas studies of the molecular mechanisms underlying plant–herbivore interactions are scarce, perhaps because herbivore experiments are difficult to conduct and control in comparison with those investigating pathogen infections. However, molecular-level research will help elucidate the mechanisms underlying herbivore responses and may provide novel insights into insect herbivory.

Most previous studies focused on plant defenses at the individual scale; however, plants normally encounter biotic attacks at the population level in the field, especially crops. Airborne signals or molecules such as VOCs, released from an attacked plant, can arouse defense responses in surrounding plants. Some VOCs even act as pest repellents. Therefore, spatiotemporal analyses of the population-level effects of such airborne signals and molecules are needed to identify potential biocontrol agents for crop protection.

SMs mediate a variety of defensive functions and enhance plant resistance to biotic attacks. It is important to understand the synthesis of these beneficial metabolites, as well as their molecular mechanisms, in the context of herbivore behavior. The application of genetically enhanced crops in SM production may enhance plant resistance. Mass production of various artificial SMs could also lead to novel strategies for the development of precision agriculture for plant management. These approaches may help mitigate crop losses under various future food demand scenarios.

## Abbreviations

**Abbreviation**	**Full Name**
AOS	Allene oxide synthase
AW	Armyworm
BPH	Brown planthopper
BABA	β-aminobutyric acid
CaMs	Calmodulins
CMLs	Calmodulin-like proteins
CCaMKs	Ca^2+^/CaM-dependent protein kinases
CDPKs/CPKs	Calcium-dependent protein kinases
CBL	Calcineurin B-like protein
CIPK	CBL interacting protein kinase
OPDA	Cis-12-oxo-phytodienoic acid
[Ca^2+^] _cyt_	Cytosolic calcium
DAMPs	Damage-associated molecular patterns
ETI	Effector-triggered immunity
ELP2	ELONGATOR PROTEIN2
GLRs	Glutamate receptor-like channels
GABA	γ-aminobutyric acid
HAMPs	Herbivore-associated molecular patterns
HATs	Histone acetyltransferases
HDACs	Histone deacetylases
HUB1	Histone monoubiquitination1
H_2_O_2_	Hydrogen peroxide
H2Bub	H2B monoubiquitination
JA	Jasmonic acid
JA-Ile	Jasmonoyl-L-isoleucine
JJW	JAV1–JAZ8–WRKY51
LOX	Lipoxygenase
LncRNAs	Long non-coding RNAs
MeJA	Methyl jasmonate
miRNAs	MicroRNAs
MAMPs	Microbe-associated molecular patterns
NADPH	Nicotinamide adenine dinucleotide phosphate
OS	Oral secretions
ACAs	P-IIB autoinhibited Ca^2+^-ATPases
ECAs	P-IIA ER-type Ca^2+^-ATPases
PRRs	Pattern recognition receptors
PTI	Pattern-triggered immunity
PAE9	Pectin acetylesterase 9
*V*m	Plasma membrane potential
RNS	Reactive nitrogen species
ROS	Reactive oxygen species
SA	Salicylic acid
SMs	Secondary metabolites
SDG	Set domain group
siRNAs	Short interfering RNAs
SAR	Systemic acquired resistance
VOCs	Volatile organic compounds
